# Laparoscopic Management of Adult Ileocolic Intussusception Secondary to an Inverted Meckel's Diverticulum

**DOI:** 10.7759/cureus.101193

**Published:** 2026-01-09

**Authors:** Tze Hee Tay, Joan Tefay, Roger Khan

**Affiliations:** 1 General Surgery, Robina Hospital, Gold Coast, AUS

**Keywords:** acute abdomen in meckel's diverticulum, adult intussusception, ileo-colic intussusception, laparoscopy, emergency laparotomy

## Abstract

Intussusception is the telescoping of one part of the bowel into an adjacent part, and it is most common in the pediatric population. In the adult population, intussusception is rare; however, it is likely secondary to a pathological lead point, such as a neoplasm. Intussusception has been described secondary to Meckel’s diverticulum, but ileocolic intussusceptions from an inverted Meckel’s diverticulum are rare. We describe here a rare case of a 33-year-old male with no past medical or surgical history presenting with an acute abdomen and imaging findings of ileocolic intussusception with bowel ischaemia. He underwent an emergency laparoscopy, where reduction was attempted but unsuccessful and an ileocolic resection was performed. Histological findings were consistent with ileocolic intussusception containing an inverted Meckel's diverticulum as a lead point. Surgical reduction and/or resection remain the mainstay of treatment when they present hemodynamically unstable and/or concerns for bowel ischaemia/perforation.

## Introduction

Intussusception is the telescoping of a segment of bowel into an adjacent bowel, and it can be categorized into entero-enteric (e.g., ileo-ileal, only confined to small bowel), colo-colic (confined to large bowel), ileocolic (ileum invaginating through ileocaecal valve), ileocaecal (where ileocaecal valve leads the intussusception), or colorectal (colon invaginating into rectum) [1]. However, the terms ileocolic and ileocaecal have been seen to be used interchangeably at times, with some articles considering intussusceptions involving the ileum and caecum to be either “ileocolic” or “ileocaecal”, whereas other articles only label it "ileocaecal" if the ileocaecal valve is the lead point. They occur most commonly in the pediatric population, and generally no anatomical anomaly is associated with their occurrence. Up to 90% of pediatric intussusceptions are ileocolic (involving the ileocaecal junction), while the remaining subtypes account for the other 10%.

In contrast, intussusceptions are rare in adults. The prevalence of ileocolic intussusceptions in adults reportedly ranges from 6% to 40% of all adult intussusceptions [1-3]. Unlike pediatric populations, abdominal pain due to intussusception in adults typically requires surgical intervention due to the higher incidence of a pathological lead point, which includes both benign (i.e. lipoma, adenomatous polyp, infective enteritis) and malignant (i.e. adenocarcinoma, lymphoma, metastatic malignancy) causes [2].

## Case presentation

**Figure 1:**
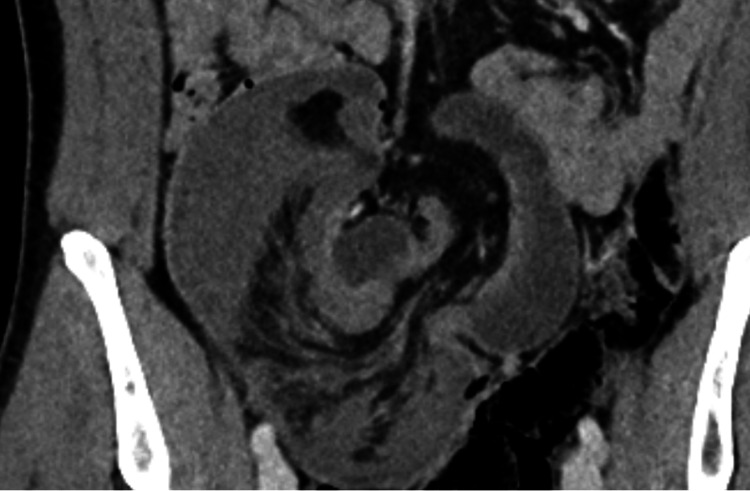
Ileocolic intussusception with hypoenhanced bowel. Coronal view of CT abdomen and pelvis, portal-venous phase.

**Figure 2:**
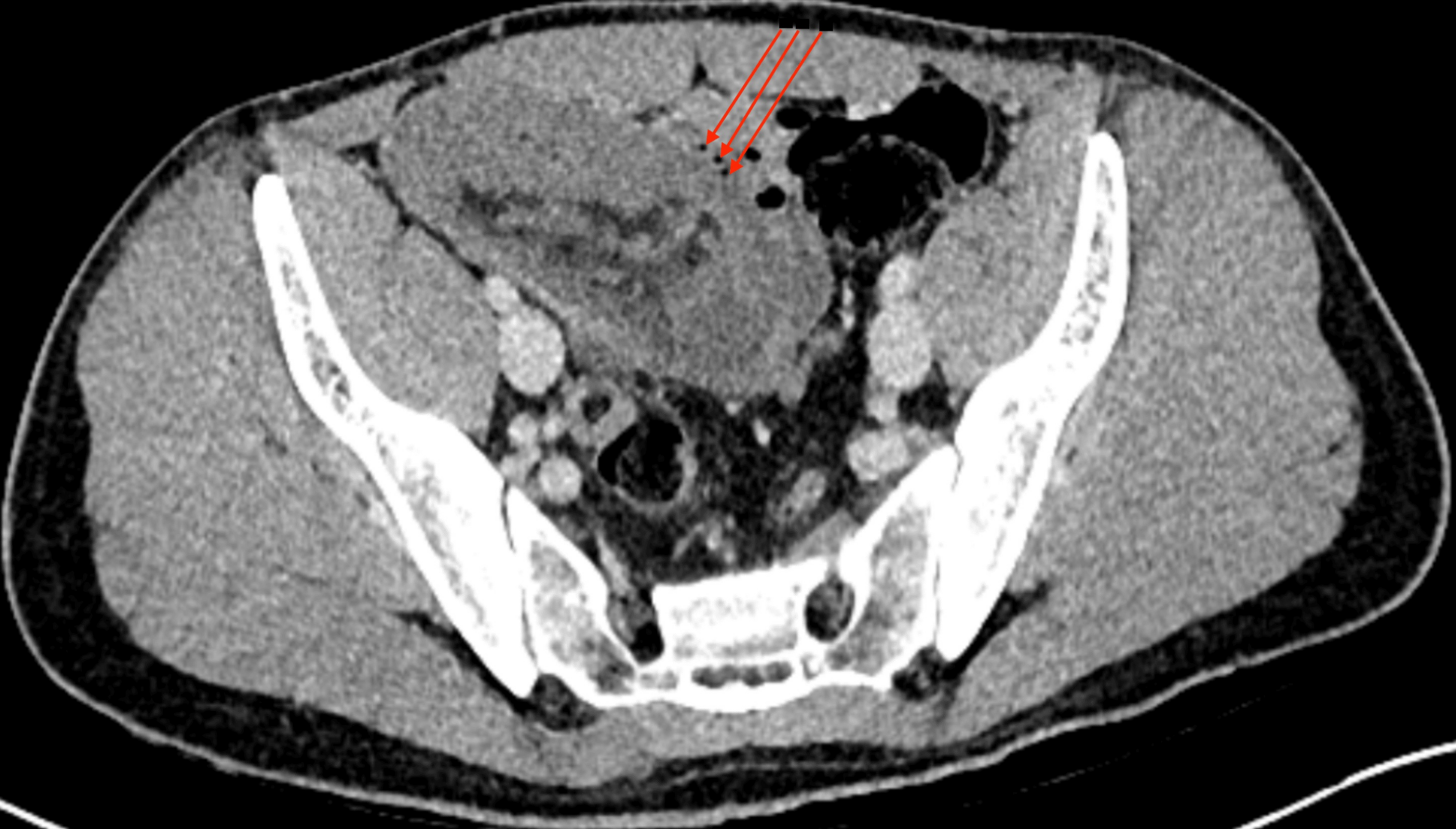
Ileocolic intussusception with hypoenhanced bowel with trace amount of extraluminal gas (red arrows). Axial view of CT abdomen and pelvis, portal-venous phase.

A 33-year-old male was transferred to the Emergency Department by ambulance due to an acute onset of severe lower abdominal pain despite parenteral opioid analgesia. On examination, he had an exquisitely tender lower abdomen and palpable mass over the right lower quadrant. Immediate venous blood gas was performed, which showed elevated lactate levels of up to 5 mmol/L. An urgent computed tomography (CT) scan was performed in the portal venous phase, which demonstrated a telescoping loop of small on large bowel in the right lower quadrant with hypoenhancement of its bowel wall, trace volume free fluid, and small locules of extraluminal gas (Figures 1-2). This is consistent with ileocolic intussusception concerning for bowel ischaemia.

**Figure 3:**
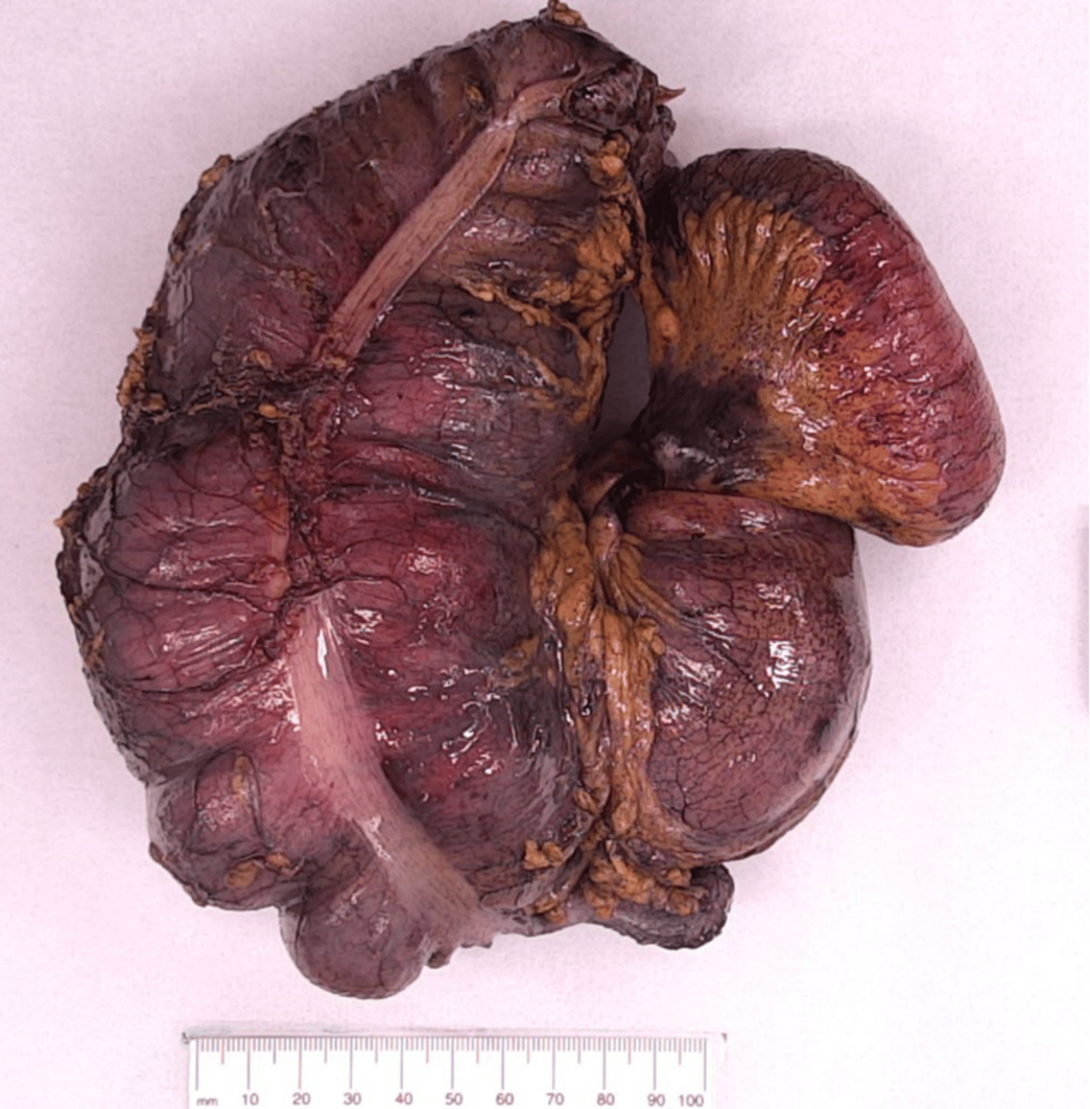
Ileocolic resection.

The patient was taken to theatre urgently for a laparoscopy, which confirmed the diagnosis of an ileocolic intussusception, forming a mass in the right iliac fossa with minimal free fluid. Laparoscopic reduction of the intussusception was attempted but was unsuccessful due to severely oedematous bowel. The decision was made to perform a laparoscopic ileocolic resection (Figure 3) with side-to-side stapled anastomosis, and a 19-french Blake's drain was placed in the right paracolic gutter. Following ileocolic resection of the intussusception, manual reduction still remained unsuccessful.

Postoperatively, he was kept on a clear fluid diet. He progressed well in the ward, and on postoperative day four, he was upgraded to a full diet, and his drain was removed. He developed post-operative ileus on day five, which was managed conservatively, and he was discharged on day 10 with outpatient follow-up.

**Figure 4:**
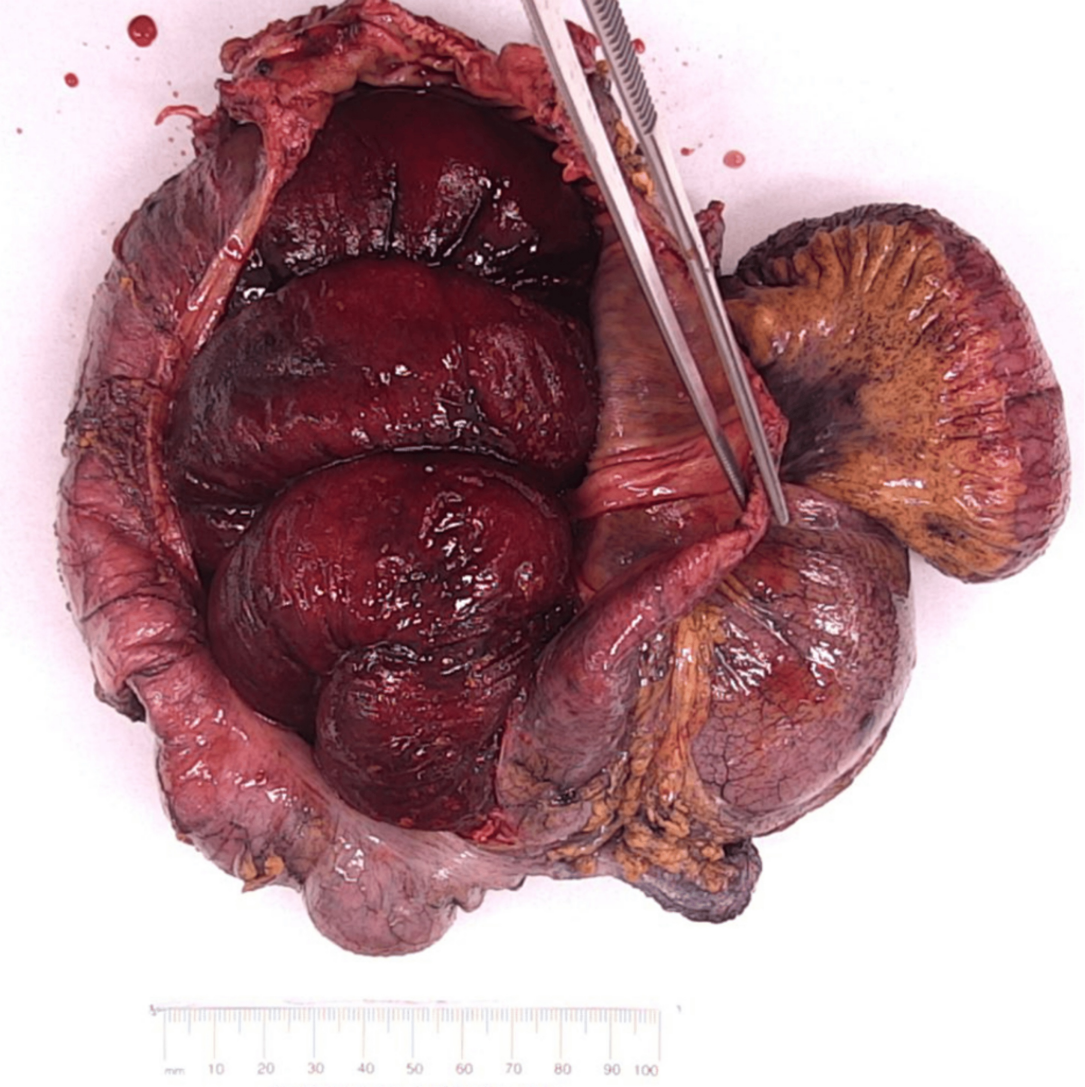
Dusky and hemorrhagic intussuscepted small bowel.

**Figure 5:**
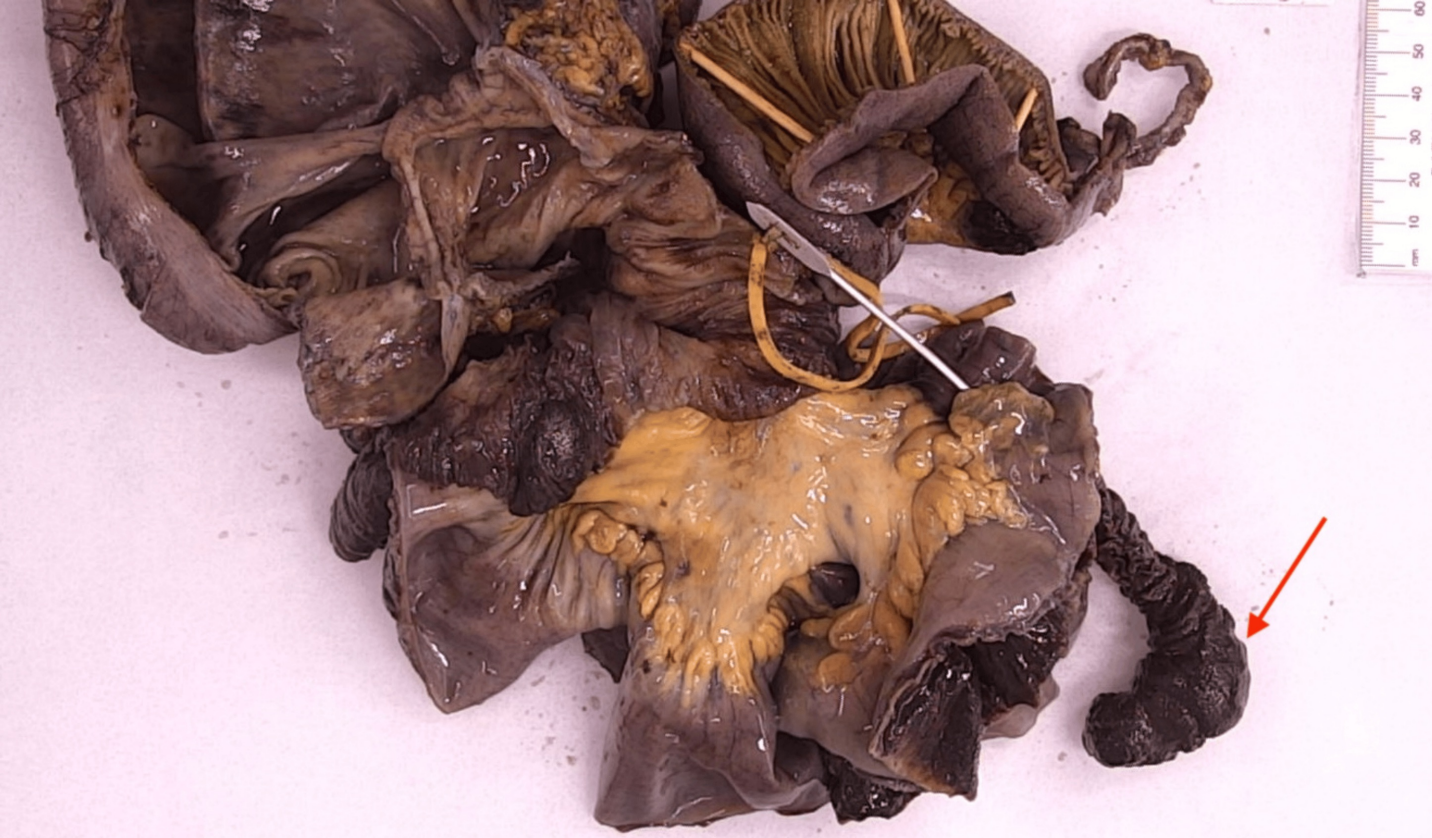
Small bowel diverticulum, red arrow.

Macroscopic examination of the intussuscepted portion of the small bowel appeared dusky and hemorrhagic (Figure 4). There is a polypoid protrusion into the small bowel lumen measuring 1 cm in length and 25 mm in diameter, which can be probed from its visceral surface, which is suspicious for an inverted diverticulum (Figure 5, red arrow). Histopathology results demonstrated that this segment of small intestine contains heterotopic gastric epithelium and pyloric glands, favouring a diagnosis of Meckel’s diverticulum.

## Discussion

Intussusception is the invagination of a portion of the proximal bowel (intussusceptum) into a distal, adjacent bowel (intussusception). This can lead to compromised venous and lymphatic drainage away from the affected bowel, which can cause congestion of the mesentery and, if left untreated, can lead to subsequent ischaemia of the affected bowel, which is the likely cause of this patient’s presentation. Sometimes, patients can present with obstructive symptoms if the intussusception completely obstructs the passage of intestinal content to cause a mechanical bowel obstruction.

Intussusceptions are mostly seen in the paediatric population without any identifiable lead point or abnormalities in the bowel/surrounding structures that tethers/obstructs the bowel [4].

Ileocolic/ileocaecal intussusception, especially in the adult population, secondary to a Meckel’s diverticulum are rare, with only nine case reports (Table 1) found to have described something similar. Out of these nine cases, only five of them are due to an inverted Meckel’s diverticulum [5-13]. All of these cases required surgical resection. 

**Table 1:** Reports of adult ileocolic/ileocaecal intussusceptions secondary to Meckel's diverticulum (MD).

Author	Year	Types of MD	Type of Intussusception	Management	Note
Steinwald et al. [5]	1996	Inverted Meckel’s diverticulum	Ileocolic	Small bowel resection	Acute abdomen - taken for emergency surgery
Lu et al. [6]	2001	Non-inverted Meckel’s diverticulum	Ileocaecal	Laparotomy, small bowel resection	First noticed on elective colonoscopy
Anastasios et al. [7]	2004	Inverted Meckel’s diverticulum	Ileocolic	Small bowel resection	Intermittent abdominal pain with normal colonoscopy 1 month prior to presentation
Tomoaki et al. [8]	2011	Inverted Meckel’s diverticulum	Ileocolic	Laparotomy, ileocolic resection (attempted reduction)	Acute abdomen - taken for emergency surgery
Sözen et al. [9]	2012	Non-inverted Meckel’s diverticulum	Ileocaecal	Open reduction and small bowel resection	Acute abdomen - taken for emergency surgery
Lafarge et al. [10]	2012	Inverted Meckel’s diverticulum	Ileocolic	Laparotomy, ileocolic resection (no reduction)	Acute abdomen - taken for emergency surgery
Soria-Céspedes et al. [11]	2015	Inverted Meckel’s diverticulum	Ileocolic	Laparotomy, ileocolic resection (no reduction)	Acute abdomen - taken for emergency surgery
Cao [12]	2019	Non-inverted Meckel’s diverticulum	Ileocolic	Laparoscopic reduction and small bowel resection	Acute abdomen - taken for emergency surgery (pregnant patient)
Hejazi et al. [13]	2022	Non-inverted Meckel’s diverticulum	Ileocaecal	Laparotomy, Ileocecectomy (no manual reduction)	Acute abdomen - taken for emergency surgery

In an otherwise healthy adult patient with an acute onset of excruciating abdominal pain requiring a cocktail of parenteral opioid analgesics, especially with convincing lactatemia on blood gas and/or obstructive symptoms concerning for a mechanical bowel obstruction, a CT scan of the abdomen is usually performed to rule out sinister causes such as perforated viscus, ischaemic bowel or mechanical small/large bowel obstruction.

Literature on the management of ileocolic intussusception in adults is sparse compared to the paediatric population, where intussusceptions are more prevalent. The aim of managing intussusception is to reduce the invagination (endoscopically or surgically) and remove any pathological lead point. In the paediatric population, reduction is usually attempted in several ways: under fluoroscopic/sonographic guidance or using hydrostatic/pneumatic reduction. However, this is because in the paediatric population, most intussusceptions present in the absence of a pathological lead point, contrary to the adult population, who are more likely to have a pathological lead point causing intussusception [4].

On literature review, there were a few cases of successful hydrostatic/pneumatic reduction of intussusception in adults. De Zoysa et al. described a case of a successful hydrostatic reduction of a right colo-colic intussusception via a rectal tube. A colonoscopy was performed a few days later, showing a caecal polyp, which was removed. Histology confirms adenocarcinoma, and the patient underwent an elective right hemicolectomy a few days later [14]. A case report published in the Journal of Minimal Access Surgery described a case of a staged approach, whereby pneumatic and hydrostatic reduction of an ileocolic intussusception was successfully performed via a colonoscopy. A large polypoid lesion was seen in the right colon, suspicious of malignancy. The patient was then taken for a laparoscopy, which showed complete resolution of the invagination, and a right colectomy was performed according to oncological principles [15]. There is also a report from 2019, which described a case of idiopathic adult intussusception of the ascending colon: initial successful reduction with colonoscopy, which showed a mass-like lesion. The patient underwent an ileocolic resection, and, interestingly, the histopathology came back negative for malignancy or other causes of intussusception [16].

Urgent surgical management must be strongly considered if there is clinical/radiological concern for bowel ischaemia and/or if there are signs of peritonism/hemodynamic instability or if non-operative reduction is unsuccessful. In this era of minimally invasive surgery, laparoscopic surgery is commonly performed in the management of adult intussusception, with similar outcomes to a laparotomy [17]. In our case, due to CT findings of hypoenhancement of the intussuscepted bowel and extraluminal gas concerning for bowel ischaemia/possible perforation, the decision was made for an emergency laparoscopy. Moreover, because of the severely oedematous bowel during laparoscopy, manual reduction was not possible, and an ileocolic resection was performed.

## Conclusions

In a case of an adult ileocolic intussusception where there is minimal concern of ischemia/perforation/hemodynamic instability, it appears that reduction by colonoscopy can be considered, in hopes of successfully reducing the invagination and assessing the bowel for causes of the intussusception. Once the cause is found, it may help with better planning of the margins of resection to avoid extensive bowel resection (if indicated). However, some authors recommend avoiding initial endoscopic reduction due to the risk of seeding of malignancy or perforation of the bowel. Furthermore, none of the above-described non-operative reduction of the intussusceptions is due to Meckel’s diverticulum. In cases when there is clinical/radiological concern for bowel ischaemia and/or if there are signs of peritonism/haemodynamic instability, surgical management with reduction (+/- resection) remains the mainstay of treatment.

Hence, with the knowledge from the current literature and our experience with this case, ileocolic intussusception from an inverted Meckel’s diverticulum generally requires surgical management with at least a laparoscopy and bowel resection.
